# Application of SNPscan in Genetic Screening for Common Hearing Loss Genes

**DOI:** 10.1371/journal.pone.0165650

**Published:** 2016-10-28

**Authors:** Zixuan Gao, Yu Lu, Jia Ke, Tao Li, Ping Hu, Yu Song, Chiyu Xu, Jie Wang, Jing Cheng, Lei Zhang, Hong Duan, Huijun Yuan, Furong Ma

**Affiliations:** 1 Department of Otolaryngology, 3^rd^ Hospital of Peking University, Beijing, China; 2 Institute of Otolaryngology, Chinese PLA General Hospital, Beijing, China; 3 Medical Genetics Center, Southwest Hospital, Third Military Medical University, Chong-qing, China; German Cancer Research Center (DKFZ), GERMANY

## Abstract

The current study reports the successful application of a fast and efficient genetic screening system for common hearing loss (HL) genes based on SNPscan genotyping technology. Genetic analysis of 115 variants in common genes related to HL, *GJB2*, *SLC26A4* and *MT-RNR*, was performed on 695 subjects with non-syndromic hearing loss (NSHL) from the Northern China. The results found that 38.7% (269/695) of cases carried bi-allelic pathogenic variants in *GJB2* and *SLC26A4* and 0.7% (5/695) of cases carried homoplasmic *MT-RNR1* variants. The variant allele frequency of *GJB2*, *SLC26A4 and MT-RNR1* was 19.8% (275/1390), 21.9% (304/1390), and 0.86% (6/695), respectively. This approach can explain ~40% of NSHL cases and thus is a useful tool for establishing primary molecular diagnosis of NSHL in clinical genetics.

## Introduction

Hearing loss (HL) is one of the most common sensorineural disorders, affecting 1–2 neonates in every 1000 and the prevalence increases with age [1]. In fact, 50% to 60% of childhood HL can be attributed to genetic defects[1]. With at least 100 genes identified to date (http://heretidaryhearingloss.org/, updated in Sep. 2016), non-syndromic HL (NSHL, with no symptoms in other organs) is an extraordinarily heterogeneous trait. Autosomal recessive (AR) NSHL is typically pre-lingual and is present in ~80% of HL cases. *GJB2* is (OMIM*121011) the most common gene involved in ARNSHL. In some populations, pathogenic *GJB2* variants account for up to 50% of all ARNSHL cases [2]. *SLC26A4* (OMIM*605646), the second most frequent gene involved in ARNSHL and is present in 4–10% of NSHL cases among different populations [3–5]. A defect in the mitochondrial gene, *MT-RNR1*, is associated with extreme sensitivity to aminoglycoside ototoxicity and accounts for maternally inherited NSHL [6, 7]. The traditional method for diagnosis of NSHL relied on Sanger sequencing. Although direct sequencing is the gold standard approach for genetic testing, it is expensive, time consuming and has low throughput. An SNPscan technique has been developed, using a patented SNP genotyping technology based on a double ligation reaction and multiplex fluorescence PCR, which is able to perform multiplex (48–192) genotyping for up to 192 kinds of SNPs[8]. This technique is uniquely suited to genetic screening for NSHL due to the extremely strong genetic heterogeneity of NSHL. In the current study, the majority of pathogenic variants of *GJB2*, *SLC26A4* and *MT-RNR1* were obtained and a primary genetic test for NSHL was designed using an SNPscan genotyping technique with the ability to simultaneously detect 115 different variants in *GJB2* (n = 36), *SLC26A4* (n = 77), and *MT-RNR1* (n = 2), the common genes related to HL.

## Materials and Methods

### Subjects and Auditory Evaluation

A total of 695 affected subjects were recruited in 2014. Patients from the Department of Otolaryngology (n = 132), 3^rd^ Hospital of Peking University (n = 132) and from 12 different rehabilitation centers for the deaf around the area of Beijing, China (n = 563) were included. This study was approved by the Ethics Committee of 3^rd^ Hospital of Peking University (Approval number 20141212). Written informed consent was obtained from adult participants and from guardians on behalf of minors/children prior to their participation in the study. A comprehensive medical history was obtained from each subject via a questionnaire regarding onset age, evolution, degree and symmetry of hearing loss, hearing aids, cochlear implants, presence of tinnitus, pressure in the ears, vertigo, history of noise exposure and other relevant clinical manifestations. Each participant received a physical examination to exclude any additional distinguishing physical findings. A comprehensive auditory evaluation, including otoscopy, pure-tone audiometry (PTA, at frequencies from 500 to 4000Hz) and immittance testing was performed. The severity of HL was defined according to PTA as mild (26-40dBHL), moderate (41–70dBHL), severe (71-90dBHL) or profound (> 90dBHL). Middle ear pressure, ear canal volume and tympanic membrane mobility was evaluated according to immittance testing.

### Screening of *GJB2*, *SLC26A4* and *MT-RNR1*

Peripheral blood samples (2 ml) were drawn from all subjects for DNA extraction and genetic analysis. Genomic DNA was isolated from whole blood using a DNA extraction kit (Axygen Scientific Inc., Union City, CA, USA). A total of 115 variants of *GJB2* (NM_004004, n = 36), *SLC26A4* (NM_000441, n = 77) and *MT-RNR1*(NR_137294, n = 2) were included. Of these, 97 pathogenic variants (*GJB2* = 18, *SLC26A4* = 77, *MT-RNR1* = 2) were pathogenic and 18 *GJB2* variants were unclassified. All the 97 pathogenic variants of *GJB2*, *SLC26A4* and *MT-RNR1* genes included in this primary screening system were taken from deafness variant database (http://deafnessvariationdatabase.org) where clear indication of the pathogenicity associated with HL were given. Screening of these pathogenic variants in common HL genes was carried out to establish molecular diagnosis for HL cases. Genetic analysis was performed using a custom-built 48-Plex SNPscanTM Kit (Genesky Biotechnologies Inc., Shanghai, China).

## Results

Based on the questionnaires, HL in 568 subjects was pre-lingual, HL in 47 subjects was post-lingual, and the age of HL onset in 80 subjects was uncertain. According to audiograms, the severity of sensorineural hearing loss was mild (n = 1), moderate (n = 15), severe (n = 69) and profound (n = 610). Thirteen subjects had a history of noise exposure and 60 subjects had a history of using aminoglycoside antibiotics (dosage uncertain), such as gentamicin and/or streptomycin. The mothers of 65 subjects suffered from possible viral infections, such as colds or urticaria, during the first 3 months of pregnancy. Comprehensive family medical histories and clinical examinations found no additional clinical abnormalities, such as cardiovascular disease, kidney disease, visual problems or neurological disorders, in any subject (Table 1).

**Table 1:** Clinical Information of 695 Chinese HL Subjects.

Onset age				Hearing loss			Environmental factors	
Pre-lingual	Post-lingual	Unknown	Mild	Moderate	Severe	Profound	Noise-exposure	Ototoxic drug
568	47	80	1	15	69	610	13	60

### *GJB2* 

#### Pathogenic variants

**Fig 1:**
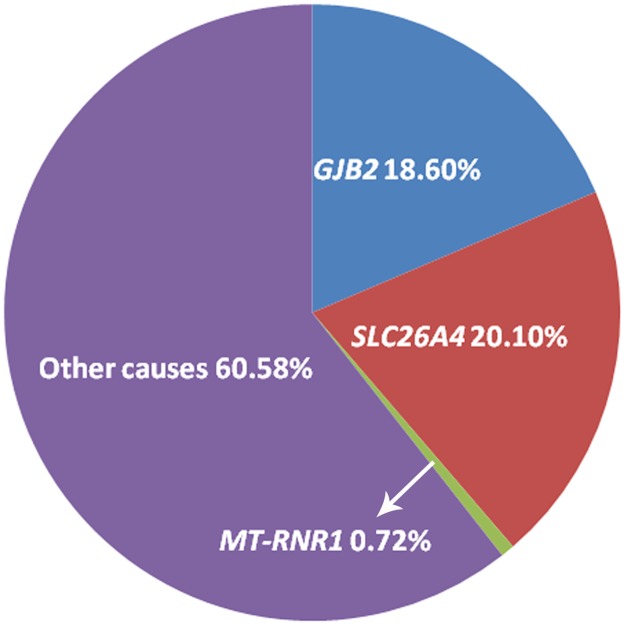
Molecular etiology of 695 Chinese patients with NSHL. The percentages of biallelic *GJB2*, *SLC26A4* and homoplasmic *MT-RNR1* pathogenic variants were 18.6%, 20.1% and 0.72%, respectively. Screening of 97 different pathogenic variants in 3 genes revealed that 129 (18.6%, 129/695) cases carried biallelic *GJB2* variants and 140 patients (20.1%,140/695) carried biallelic *SLC26A4* variants. Homoplasmic *MT-RNR1* m.1555A > G occurred in 5 cases (0.72%,5/695), however, *MT-RNR1* variants are not necessarily causative for HL.

**Fig 2:**
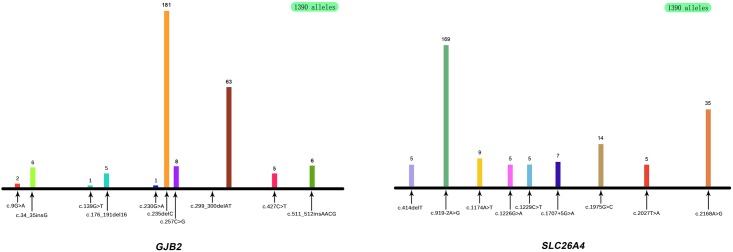
Location of pathogenic variants of *GJB2* and *SLC26A4* in the 695 subjects with NSHL. (a)Overall, 11 different *GJB2* pathogenic variants were identified in 695 subjects, out of which, one subject carried a heterozygous c.IVS1+1G > A mutant allele. The number of the mutant alleles of the other 10 different pathogenic variants, c.9G>A, c.34_35insG, c.139G>T, c.176_191del16, c.230G>A, c.235delC, c.257C>G, c.299_300delAT, c.427C>T and c.511_512insAACG are shown here. (b) In addition, 41 *SLC26A4* pathogenic variants were identified in 695 subjects, the number of the mutant alleles of the most frequent 9 variants, c.414delT, c.919-2A>G, c.1174A>T, c.1226G>A, c.1229C>T, c.1707+5G>A, c.1975G>C, c.2027T>A and c.2168A>G are shown here.

Genetic analysis revealed that 17 subjects carried only one *GJB2* mutant allele and 129 (18.6%, 129/695) subjects carried bi-allelic pathogenic variants in *GJB2*, including 57 homozygotes and 72 compound heterozygotes (Fig 1). Therefore, the allele frequency of pathogenic variants in *GJB2* gene was 19.8% (275/1390). The allele frequency of c.235delC, the most prevalent pathogenic variant of *GJB2*, was 13.0% (181/1390), followed by c.299_300 del AT, c.257C > G, c.34_35insG and c.511_512insAACG with allele frequencies of 4.5% (63/1390), 0.58% (8/1390), 0.43% (6/1390) and 0.43% (6/1390), respectively. These 5 pathogenic variants account for 96.0% (264/275) of all *GJB2* mutant alleles. In addition, c.235delC was a compound heterozygote, with 9 different pathogenic variants, in 73 patients and was present as a mono-allelic variant in 2 patients who carried bi-allelic pathogenic variants of *SLC26A4*. Overall, 11 pathogenic variants of *GJB2* (Fig 2) constituted 14 different genotypes (Table 2).

**Table 2:** Chinese Patients with Bi-allelic Pathogenic variants in *GJB2*.

Genotypes	Number (%) of patients	Inheritance mode	Clinical information	
			HL	Onset age (Years)
**Homozygous**				
c.235delC/ c.235delC	52 (7.48%)	AR *	Severe, profound	Did not pass NBHS~ 13
c.299_300delAT/ c.299_300delAT	5 (0.72%)	AR	Profound	3 months~3.5
**Compound heterozygous**				
c.235delC/c.299_300delAT	40 (5.76%)	AR	Severe, profound	6 months~3.5
c.235delC/c.34_35insG	6 (0.86%)	AR	Severe, profound	1~5
c.235delC/c.511_512insAACG	6 (0.86%)	AR	Severe, profound	9 months~5
c.235delC/c.257C>G	4 (0.58%)	AR	Severe, profound	6months~3
c.235delC/c.427C>T	4 (0.58%)	AR	Severe, profound	11 months~4
c.235delC/c.176_191del16	3 (0.43%)	AR	Profound	1~2.5
c.299_300delAT/c.257C>G	3 (0.43%)	AR	Severe, profound	Did not pass NBHS ~2
c.235delC/c.9G>A	2 (0.29%)	AR	Severe, profound	1~3
c.176_191del16/c.230G>A	1 (0.14%)	AR	Profound	1.5
c.176_191del16/c.139G>T	1 (0.14%)	AR	Profound	4
c.235delC/c.IVS1+1G>A	1 (0.14%)	AR	Severe	1
c.299_300delAT/c.427C>T	1 (0.14%)	AR	Severe	6 months
Total	129 (18.6%)			

*AR: Autosomal recessive

#### Unclassified variants

Genetic analysis of 18 unclassified *GJB2* variants revealed that 7 variants in 50 cases. Specifically, 3 cases carried bi-allelic variants (one c. 95G > A homozygote and 2 c.109G > A homozygotes) and 47 cases carried 1 *GJB2* variant. The allele frequency of c.109G > A was 3.2% (44/1390) and c.95G > A, c.283G > A, and c.571T > C shared the same allele frequency of 0.14% (2/1390). Moreover, c.23C > T, c.408C > A and c.416G > A occurred in one case each as a heterozygous variant, sharing an allele frequency of 0.07% (1/1390) (Tables 3 and 4).

**Table 3:** Unclassified *GJB2* variants in Patients with NSHL.

Variants	Number of patients	Clinical information	
		HL	Onset age (Years)
**Homozygous**			
c.95G>A/ c.95G>A	1	Profound	Unclear
c.109G>A /c.109G>A	2	Mild	3
		Profound	6 months
**Heterozygous**			
c.23C>T	1	Severe	1
c.109G>A	40	Moderate-profound	Did not pass NBHS*~4
c.283G>A	2	Severe-profound	6 months~5
c.408C>A	1	Profound	5
c.416G>A	1	Severe	6
c.571T>C	2	Severe, profound	6 months ~2
Total	50		

*NBHS: Newborn hearing screening

**Table 4:** The allele frequency of Unclassified *GJB2* variants in Patients with NSHL.

Variants	Number (%)of mutant alleles	Allele frequency in Database		
		ExAC(East Asian)	1000 genomes(Han Chinese in Beijing)	ClinVar
c.23C>T	1(0.07%)	0%	0%	-
c.95G>A	2(0.14%)	0%	-	-
c.109G>A	44(3.2%)	7.2%	3.9%	GO-ESP 0.13%; GMAF 1.5%
c.283G>A	2(0.14%)	0%	0%	GMAF 0.02%
c.408C>A	1(0.07%)	0%	-	-
c.416G>A	1(0.07%)	0.01%	0.49%	GO-ESP 0.054%; GMAF 0.04%
c.571T>C	2(0.14%)	0.2%	0.49%	GMAF 0.02%

-: no record

### *SLC26A4* 

A total of 164 patients (23.6%, 164/695) had molecular defects in the *SLC26A4* gene and 140 patients (20.1%, 140/695) carried bi-allelic pathogenic variants in *SLC26A4* (Fig 2), including 54 homozygotes and 86 compound heterozygotes. Twenty-four cases carried a heterozygous *SLC26A4* mutant allele and 3 of these also carried bi-allelic pathogenic variants of *GJB2*. Thus, the mutant allele frequency of *SLC26A4* was 21.9% (304 /1390). Genetic analysis of *SLC26A4* revealed 52 different genotypes (Table 5) comprised by 41 *SLC26A4* variants (Table 6). The 5 most prevalent pathogenic variants in *SLC26A4* were c.919-2A > G (12.2%, 169/1390), c.2168A > G (2.5%, 35/1390), c.1975G > C (1.0%,14/1390), c.1174A > T (0.65%,9/1390) and c.1707+5G > A(0.5%, 7/1390), and accounted for 77.0% (234/304) of all mutant *SLC26A4* alleles(Fig 2). The most prevalent pathogenic variant, c.919-2A > G, accounting for 55.6% (169/304) of all *SLC26A4* mutant alleles, was in compound heterozygosity with 24 different *SLC26A4* variants in 60 cases.

**Table 5:** Chinese Patients with Bi-allelic Pathogenic variants in *SLC26A4*.

Genotypes	Number (%) of patients	Inheritance mode	Clinical information	
			HL	Onset age(Years)
**Homozygous**				
c.919-2A>G/c.919-2A>G	50 (7. 19%)	AR *	Moderate, severe, profound	Did not pass NBHS~ 8
c.414delT/c.414delT	2 (0. 29%)	AR	Profound	5 months, 1
c.1707+5G>A/c.1707+5G>A	1 (0. 14%)	AR	Profound	2
c.2168A>G/c.2168A>G	1 (0. 14%)	AR	Profound	2
**Compound heterozygous**				
c.919-2A>G/c.2168A>G	17 (2. 44%)	AR	Moderate, severe, profound	6 months ~11
c.919-2A>G/c.1975G>C	9 (1. 29%)	AR	Severe, profound	2 ~3. 5
c.919-2A>G/c.1174A>T	3 (0. 43%)	AR	Severe, profound	2~3
c.919-2A>G/c.1226G>A	3 (0. 43%)	AR	Profound	2~3
c.919-2A>G/c.1707+5G>A	3 (0. 43%)	AR	Severe, profound	8months~2
c.919-2A>G/c.279T>A	2 (0. 29%)	AR	Profound	2, 4
c.919-2A>G/c.754T>C	2 (0. 29%)	AR	Profound	2, 7
c.919-2A>G/c.916_917insG	2 (0. 29%)	AR	Profound	1. 5, 3
c.919-2A>G/c.1225C>T	2 (0. 29%)	AR	Severe, profound	1, 2
c.919-2A>G/c.1229C>T	2 (0. 29%)	AR	Profound	2, 4
c.2168A>G/c.1174A>T	2 (0. 29%)	AR	Severe, profound	2, 3. 5
c.919-2A>G/c.1343C>T	2 (0. 29%)	AR	Severe, profound	2~3
c.2168A>G/c.1927G>T	2 (0. 29%)	AR	Moderate, severe	1, 1
c.2168A>G/c.227C>T	1 (0. 14%)	AR	Severe	1
c.235C>T/c.1594A>C	1 (0. 14%)	AR	Profound	2
c.249G>A/c.1707+5G>A	1 (0. 14%)	AR	Severe	2. 5
c.269C>T/c.1229C>T	1 (0. 14%)	AR	Severe	4
c.281C>T/c.919-2A>G	1 (0. 14%)	AR	Severe	2
c.281C>T/c.1174A>T	1 (0. 14%)	AR	Profound	2
c.414delT/c.919-2A>G	1 (0. 14%)	AR	Profound	1
c.439A>G/c.2027T>A	1 (0. 14%)	AR	Profound	3
c.563T>C/c.919-2A>G	1 (0. 14%)	AR	Profound	2
c.563T>C/c.2168A>G	1 (0. 14%)	AR	Profound	12
c.589G>A/c.2168A>G	1 (0. 14%)	AR	Profound	1. 5
c.754T>C/c.1226G>A	1 (0. 14%)	AR	Profound	6
c.916_917insG/c.1975G>C	1 (0. 14%)	AR	Severe	1
c.919-2A>G/c.946G>T	1 (0. 14%)	AR	Profound	2
c.919-2A>G/c.1079C>T	1 (0. 14%)	AR	Profound	2
c.919-2A>G/c.1173C>A	1 (0. 14%)	AR	Severe	3
c.919-2A>G/c.1340delA	1 (0. 14%)	AR	Profound	4
c.919-2A>G/c.1343C>A	1(0. 14%)	AR	Severe	2
c.919-2A>G/c.1371C>A	1 (0. 14%)	AR	Profound	1
c.919-2A>G/c.1489G>A	1 (0. 14%)	AR	Profound	3
c.919-2A>G/c.1547_1548InsC	1 (0. 14%)	AR	Severe	1
c.919-2A>G/c.1586T>G	1 (0. 14%)	AR	Moderate	10
c.919-2A>G/c.1673A>T	1 (0. 14%)	AR	Severe	3
c.1174A>T/c.1229C>T	1 (0. 14%)	AR	Severe	1
c.1225C>T/c.1594A>C	1 (0. 14%)	AR	Profound	1
c.1226G>A/c.2168A>G	1 (0. 14%)	AR	Profound	1. 5
c.1229C>T/c.2027T>A	1 (0. 14%)	AR	Profound	6 months
c.1327G>C/c.2168A>G	1 (0. 14%)	AR	Profound	1
c.1334T>G/c.1547_1548InsC	1 (0. 14%)	AR	Profound	9 months
c.1340delA/c.2027T>A	1 (0. 14%)	AR	Profound	2
c.1343C>T/c.1975G>C	1 (0. 14%)	AR	Severe	3
c.1686_1687insA/c.2168A>G	1 (0. 14%)	AR	Profound	1
c.1686_1687insA/c.2027T>A	1 (0. 14%)	AR	Severe	2. 5
c.1707+5G>A/c.1975G>C	1 (0. 14%)	AR	Profound	3
c.2027T>A/c.2168A>G	1 (0. 14%)	AR	Severe	4
Total	140 (20. 1%)			

*AR: Autosomal recessive

**Table 6:** Recessive Pathogenic Variants in *SLC26A4* in Patients with NSHL.

Base pair change	Effect or Amino acid change	TM* domain	Number of subjects	Number of mutant alleles
c.147C>G	Ser49Arg	NH2	2	2
c.227C>T	Pro76Leu	NH2	1	1
c.235C>T	Arg79Ter	NH2	1	1
c.249G>A	Trp83 Ter	NH2	1	1
c.269C>T	Ser90Leu	Exon 1	1	1
c.279T>A	Ser93Arg	Exon 1	2	2
c.281C>T	Thr94Ile	Exon 1	3	3
c.414delT	Frame-shift	Exon 3	3	5
c.439A>G	Met147Val	Exon 3	1	1
c.563T>C	Ile188Thr	Exon 4	2	2
c.589G>A	Gly197Arg	Exon 4	1	1
c.754T>C	Ser252Pro	EC LOOP3	3	3
c.916_917insG	Frame-shift	Exon 7	3	3
c.919-2A>G	Splice site	None	119	169
c.946G>T	Gly316 Ter	Exon 7	1	1
c.1079C>T	Ala360Val	Exon 8	1	1
c.1173C>A	Cys391 Ter	Exon 9	2	2
c.1174A>T	Asn392Thr	Exon 9	9	9
c.1225C>T	Arg409Cys	EC LOOP5	3	3
c.1226G>A	Arg409His	EC LOOP5	5	5
c.1229C>T	Thr410Met	EC LOOP5	5	5
c.1327G>C	Glu443Gln	IC LOOP5	1	1
c.1334T>G	Leu445Trp	IC LOOP5	1	1
c.1336C>T	Gln446 Ter	IC LOOP5	1	1
c.1340delA	Frame-shift	IC LOOP5	2	2
c.1343C>A	Ser448 Ter	IC LOOP5	1	1
c.1343C>T	Ser448Leu	IC LOOP5	3	3
c.1371C>A	Asn457Lys	Exon 11	1	1
c.1489G>A	Gly497Ser	Exon12	1	1
c.1547_1548InsC	Frame-shift	COOH	2	2
c.1586T>G	Ser529Ala	COOH	1	1
c.1594A>C	Ser532Arg	COOH	2	2
c.1673A>T	Asn558Ile	COOH	1	1
c.1686_1687insA	Frame-shift	COOH	2	2
c.1707+5G>A	Splice site	COOH	6	7
c.1927G>T	Glu643 Ter	COOH	2	2
c.1975G>C	Val659Leu	COOH	14	14
c.1991C>T	Ala664Val	COOH	1	1
c.2027T>A	Leu676Gln	COOH	5	5
c.2167C>G	His723Asp	COOH	1	1
c.2168A>G	His723Arg	COOH	34	35
**Total**			164	304

*TM: Transmembrane domains

### *MT-RNR1* pathogenic variants

**Fig 3:**
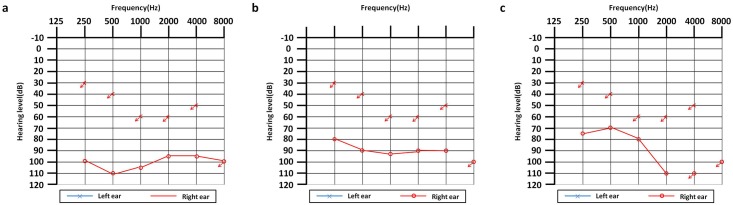
Patients with characteristic audiograms. (a) One case (female, 4 years old, onset at birth) with bi-allelic pathogenic variants in *GJB2* had a cochlear implant in the left ear and had profound hearing loss in the right ear. (b) One case (male, 3 years old, onset at 1 year old) with bi-allelic pathogenic variants in *SLC26A4* had a cochlear implant in the left ear and had severe hearing loss in the right ear. (c)One case (male, 3 years old, onset at 2 yrs) with homoplasmic *MT-RNR1* m.1555A > G had a cochlear implant in the left ear and had severe hearing loss in the right ear with a down-sloping shaped audiogram, which is characteristic of aminoglycoside-induced HL.

*MT-RNR1* m.1555A > G occurred in 6 patients (5 homoplasmons and 1 heteroplasmon). The mutant allele frequency of *MT-RNR1* was 0.86% (6/695). The percentage of homoplasmic *MT-RNR1* pathogenic variants was 0.72% (5/695) (Table 7). Of the 5 cases with homoplasmic variants, 1 case had normal hearing, and then was found to be hard of hearing at 19 months, 2 weeks after the use of amikacin and 1 case showed characteristic down-sloping high-frequency hearing loss (Fig 3). *MT-RNR1* m.1494C > T occurred in no subjects.

**Table 7:** HL cases with *MT-RNR1* m.1555A>G.

Number	Age (years)	Onset age(years)	Hearing loss	History of aa*	m. 1555A>G
1	5	19 months	All frequencies, profound	Amikacin	Homo
2	3	1	All frequencies, profound	Unclear	Homo
3	22	Unclear	All frequencies, profound	Unclear	Homo
4	3	2	All frequencies, profound	Unclear	Homo
5	3	2	High frequencies. severe	Unclear	Homo
6	7	3 days	All frequencies, profound	Unclear	Hetero

*Aa: aminoglycoside antibiotics

## Discussion

### *GJB2, SLC26A4* 

The *GJB2* gene, encoding gap junction protein Connexin 26(CX26), is the most frequent causative gene for NSHL. In this study, bi-allelic *GJB2* pathogenic variants accounted for 18.6% of HL among the 695 subjects in this study. This is comparable with the previously reported frequency of 17.9% in 2063 Chinese HL patients [9]. According to previous reports, pathogenic variants of c.35delG, c.167delT, and c.235delC are the 3 most frequent pathogenic variants in Caucasian, Ashkenazi Jewish, and Asian populations [10–14]. The frequency of c.235delC varies from 4% to 30.4% among different regions in China [9]. In this study, *GJB2* c.235delC was the most common pathogenic variant with a frequency of 13.0% (181/1390). Moreover, the panel in this study contained 18 unclassified *GJB2* variants with undetermined pathogenicity and 7 of these variants were detected in 50 of the 695 cases examined. The allele frequencies of c.109G > A, c.95G > A, c.283G > A, c.571T > C, c.23C > T, c.408C > A and c.416G > A were 3.2%, 0.14%, 0.14%, 0.14%, 0.07%, 0.07%, and 0.07%, respectively. It’s important to know that the pathogenic association between these variants and HL is uncertain and therefore the 3 subjects (one c.95G > A and 2 c.109G > A homozygotes) cannot assumed to be deaf or hard of hearing on the basis of having variants of unknown significance (VUS). The significance of this primary screening system was to collect subjects with VUSs; further studies must be done to investigate the possibility of a causative relationship between these variants and HL.

*SLC26A4* (OMIM* 605646) plays a key role in maintaining the endo-cochlear potential and is the second most common causative gene for ARNSHL [3–5]. According to a previous study, *SLC26A4* bi-allelic pathogenic variants account for approximately 11% of Chinese Han probands with severe-to-profound HL and analysis of *SLC26A4* in 176 unrelated Chinese patients with ARNSHL demonstrated that 13.6% (24/176) of patients carried at least one mutant allele [15]. In this study, 23.6% patients were found to have genetic defects in *SLC26A4*, out of which, 20.1% carried bi-allelic pathogenic variants and 3.5% carried one mutant allele. The 5 most prevalent pathogenic variants, c.919-2A > G, c.2168A > G, c.1975G > C, c.1174A > T and c.1707+5G > A, are detected in 77.0% of all mutant *SLC26A4* alleles.

Sensorineural hearing loss has been described as a monogenic disease, encompassing bi-allelic pathogenic variants in patients with ARNSHL. Genetic testing has previously been completed and a final molecular diagnosis has been made upon the identification of bi-allelic pathogenic variants within one gene. Inherited as a classic Mendelian trait, typical ARNSHL is caused by bi-allelic pathogenic variants in one gene, giving rise to a 25% risk of ARNSHL in the siblings of an HL subject. However, in the current study, 2 independent genes were involved in HL, indicating an increased risk prediction for siblings and further offspring of patients. In this study 3 patients carried bi-allelic *GJB2* variants and heterozygous *SLC26A4* variants and 2 patients carried bi-allelic *SLC26A4* variants and heterozygous *GJB2* variants. Simultaneous analysis of common HL genes yields more accurate genetic findings related to HL and has vital implications for improving genetic counseling and risk prediction. Furthermore, it’s important to recognize that it cannot be assumed that having pathogenic variants in more than 1 gene is equivalent to di-genic inheritance. Without functional evidence, the conclusion for di-genic inheritance cannot be made.

### *MT-RNR1* 

The mitochondrial gene, *MT-RNR1* encodes 12S ribosomal RNA and is considered to be a chief cause for non-syndromic HL and aminoglycoside-induced HL [16]. Homoplasmic m.1555A > G and m.1494C > T, located at the highly conserved decoding site of the 12S ribosomal RNA gene, have been identified as a cause of maternally inherited non-syndromic HL. HL inherited through the mitochondria accounts for less than 1% of all HL, though the frequency of m.1555A > G in Chinese non-syndromic HL patients can vary from 1.67%-15.5% [17–20]. In this study, 5 cases (0.72%,5/695) were found to carry homoplasmic m.1555A > G, out of which, 1 had a history of aminoglycoside antibiotic usage and another had a recognizable audio-profile of aminoglycoside-induced HL. Thus, *MT-RNR1* m.1555A > G is one possible reason for hearing loss in these 2 cases. However, it is important to recognize that even though *MT-RNR1* m.1555A > G is not necessarily causative for HL, mitochondrial genes are transmitted maternally and therefore, identification of *MT-RNR1* m.1555A > G and m.1494C > T may have substantial implications for genetic counselling and offer an early warning for maternal members in the family pedigree.

### SNPscan technique

Single nucleotide polymorphisms (SNPs), the third generation of DNA genetic markers after Microsatellites, have been widely used in molecular genetics. In the current study, multiplex genotyping of 115 variants in 695 patients was successfully performed using an SNPscan technique developed by our team. It is a rapid and efficient molecular diagnostic strategy for identifying common genes related to deafness in Chinese populations and could be used clinically as a primary screening system, leading to advances in terms of power and cost efficiency.

This screening approach has been confirmed using Next Generation Sequencing and unpublished data from our lab showed that 18 pathogenic *GJB2* variants and 77 pathogenic *SLC26A4* variants in our panel detected 98% of all known *GJB2* and 96% of all known *SLC26A4* mutant alleles in our cohort. SNPscan analysis demonstrated that the cause of hearing loss in 61.3%(425/695) of subjects was unidentified; further analysis of all known genes related to deafness will be carried out using targeted genomic enrichment (TGE) and massively parallel sequencing (MPS).
